# On the Treatment of Rheumatic Fever

**Published:** 1864-04

**Authors:** J. Birbeck Nevins

**Affiliations:** Lecturer on Materia Medica, Royal Infirmary School of Medicine, Liverpool


### ON THE TREATMENT OF RHEUMATIC FEVER.

By Dr. J. BIRBECK NEVINS, Lecturer on Materia Medica, Royal Infirmary School of Medicine, Liverpool.

[The plan of treatment recommended by Dr. Nevins is one which he has tried extensively for above fifteen years, though he does not claim the credit of originality. During this period he has also tried the various plans in vogue, viz., the opiate, the alkaline, the lemon-juice, and the do-nothing treatment, but he has always returned to his accustomed plan.]

It is impossible to observe many cases of rheumatic fever without being struck by the periodicity of the disease, as shown by the general aggravation of the pain and other symptoms as night comes on, and also by the copious sweating which enfeebles the patient, rather than relieves him. The long continuance of the illness, and its liability to return after apparent recovery, and the length of time requisite for regaining strength, are also well known features. In some of these particulars, but especially in its periodical exacerbations and in its sweatings, Heberden and others, and Dr. Davis, of University College, in a very able paper on the subject, have at different times noted its similarity to ague, and advocated the employment of cinchona or quinine for its cure; and it is this drug upon which I look as the basis of the treatment to be proposed to you. At the same time the experience of the profession generally has shown the great value of iodide of potassium in chronic rheumatism; and, remembering the tendency of this disease to become chronic, I always combine this medicine with the quinine, and commence their administration *from the earliest date at which the patient comes under my care.* The presence of actute pain and high febrile excitement does not, in my experience, form any objection to their employment; and the thick creamy fur upon the tongue disappears more rapidly under their use than under the different methods which I have compared with it, in my own practice, or when noticing that of my brethren in the profession. The dose never exceeds two grains of quinine four times a day, with five grains of iodide of potassium added to each dose.

The pain and loss of rest are, however, so distressing to the patient, that we have been advised to administer opium in quantities only limited by the effect produced. And the employment of this drug as far as may be necessary for subduing the pain is a very important point; and I therefore always leave two or three doses of opium pill or of Dover's powder with the nurse, which are to be given successively, if the patient is in severe pain; but I very rarely indeed find that the patient has even asked for more than a single dose in the twenty-four hours, which I attribute to the speedy and more permanent relief obtained by the followlng element of the treatment, to which I attach very great importance. This is, *the employment from the very first of steam-baths, even when the patient is so helpless that it is impossible to move him from the bed* on which he is lying. These steam-baths relieve the pain and check the distressing perspirations in a degree which I have failed to obtain by any other mode of treatment; and they are administered with the greatest ease in the following manner:—

A couple of common red bricks are to be placed in an oven hot enough for baking bread, and in half an hour or little more they are sufficiently heated for the purpose. The patient's body-linen having been previously removed, these two bricks are to be folded up in a piece of common thick flannel thoroughly soaked in vinegar and laid upon two plates; and one is to be placed about a foot distant from one shoulder, and the other about equally distant from the opposite leg;* and the bedclothes are then to cover the bricks and the patient closely round his neck. A most refreshing acid steam-bath is thus obtained; and the supply of steam may be kept up, if necessary, by removing one brick and replacing it with another hot one kept in reserve. When the patient has been in the bath for about fifteen or twenty minutes, the bedclothes and plates should be removed, and *the patient instantly mopped all over very rapidly with a towel wrung out of cold water*, and then should be quickly rubbed dry.† Dry warm linen must be put on at once, and dry bedclothes must replace those which were on the bed

*Care must be taken not to put the bricks too near the body. I have known the thigh blistered in a patient who was unable to move away from the heat which was accidentally very near it. A dry napkin thrown over the wetted one will prevent this accident, if the bed is too narrow to allow sufficient space.

†The under sheet can be removed, and a dry one substituted by fastening the corners of the dry sheet to those of the damp one. Very little difficulty is generally met with in simply drawing the old sheet from under the patient, when the dry one follows it, and is left in its place.

previously. The patient generally experiences great and speedy relief from this bath. The exhausting acid sweats are materially diminished; and the necessity for opium, as already mentioned, is almost at an end.

But here the objection naturally presents itself: a patient in rheumatic fever suffers so severely from the slightest attempt to move him, that we are frequently obliged to leave him several days without changing his linen, from the pain occasioned by the attempt to remove it even leisurely; and we have just been told to change it quickly, which implies that the case cannot be a very severe one, or this direction could not be carried out. The difficulty is really of the most trifling character, if the simple precaution is adopted of tearing the night-shirt open from top to bottom down the back. The sleeves are then slipped over the patient's arms almost without moving them; and the torn edges of the linen are gently tucked under his side, from which they can be just as easily withdrawn the next day. And by this means he is freed from the discomfort of lying day after day in linen soaked with acid perspiration; and this is done without the smallest pain to himself or trouble to his nurse. For many years I used large lumps of quicklime, and wrapped them up in cloths soaked with cold water; and as soon as the lime began to slack, the patient was enveloped in a steam-bath from simple water; but in many places it is difficult to obtain quicklime, and the vinegar is also more refreshing to the patient; so that the vinegar and hot bricks have now quite superseded the lime-bath.

These, then, are *the essentials of the treatment: quinine and iodide of potassium from the first, and the steam-bath, with the subsequent cold sponging;* and as an adjunct, opium in small doses, when necessary, to procure sleep.

It now remains to speak about the success of the treatment. During the fifteen years it has been in use, I have only had occasion to apply a blister over the heart in three instances; and this was done because the patient complained of uneasiness in the chest, not because there was any distinct evidence of pericarditis. There has not been one case of distinct rheumatic affection of the heart; but the absence of clinical reports puts it out of my power to state how many cases have been thus treated. I can merely say that they have been numerous.

Next, as regards the duration of the disease; it is extremely rare that it is necessary to give two-steam-baths in bed, the patient being almost always able to have the seeond whilst sitting upon a chair; from which you will draw your own conclusion as to the rapidity of improvement. I am surprised when the patient is not able to walk about the room, a little at any rate, in little more than a week; and I have a strong impression that he is more frequently able to do this within the week than not. But here, again, the abscence of exact reports must be taken into account. I further think that from two to three weeks is the average duration of the case before the patient is able to walk up and down stairs, and to go out of doors for exercise or pleasure. Relapses are not common; and the patient has not the lingering convalescence which I have observed under other methods of treatment.

The steam-baths and subsequent cold douche should be continued after the patient is able to walk about, as they contribute to the healthy action of the skin, and promote the free mobility of the joints.*

If there is great tenderness of any one particular joint, an opiate embrocation, containing in addition either chloroform or tincture of aconite should be gently painted over the part two or three times a-day; but, in the early stage, the employment of friction appears unadvisable whilst the pain is very acute.

The recommendations of the method now presented to you are: that the patient's strength is husbanded from the first, and he has neither the protracted disease nor the lingering convalescence often observed. Pain and sweating are more quickly relieved than by any other treatment I have seen. Relapses are very rare; and so far I have not seen any case of cardiac affection occurring as a consequence of the rheumatism. I have a strong conviction that, if the method is fairly used in two or three cases, it will leave the same favorable impression upon the minds of those who try it that it has produced upon my own, and upon the students who have watched its employment in the hospital to which I have alluded.—*British Medical Journal, August* 1, 1863, *p.* 113.

*These baths are very easily given by placing the patient naked upon a chair, and putting a can containing a couple of gallons of boiling water under it. Blankets are then to be folded round his neck, and made to surround him like a tent, reaching to the floor. In about five or ten minutes, a red-hot brick should be put into the can, which renews the supply of steam. The patient soon perspires; and in fifteen or twenty minutes the blankets should be removed, and a couple of quarts of cold water should be poured over his shoulders: or, if he is afraid of such heroic treatment, he should be mopped from head to foot with towels wrung out of cold water. By this means he is invigorated instead of feeling weakened, and depressing perspirations do not follow the bath. The patient should sit upon a pillow or doubled blanket, on a close-bottomed chair, not upon an open cane-bottomed one. I have known a patient scalded by the accidental neglect of this precaution.

